# Cyclic phosphonium ionic liquids

**DOI:** 10.3762/bjoc.10.22

**Published:** 2014-01-24

**Authors:** Sharon I Lall-Ramnarine, Joshua A Mukhlall, James F Wishart, Robert R Engel, Alicia R Romeo, Masao Gohdo, Sharon Ramati, Marc Berman, Sophia N Suarez

**Affiliations:** 1 Chemistry Department, Queensborough Community College of the City University of New York, 222-05 56th Avenue, Bayside, NY, 11364, USA, Fax: +1 718-281-5078, Tel: +1 718-281-5572; 2 Department of Chemistry and Biochemistry, Queens College of the City University of New York, 65-30 Kissena Boulevard, Flushing, NY, 11367, USA, Fax: +1 718-997-5531, Tel: +1 718-997-4106; 3 Chemistry Department, Brookhaven National Laboratory, Upton, NY, 11973, USA, Fax: +1 631-344-5815, Tel: +1 631-344-4327; 4 The Institute of Scientific and Industrial Research (ISIR), Osaka University, 8-1 Mihogaoka, Ibaraki, Osaka 567-0047, Japan; 5 Department of Physics and Astronomy, Hunter College of the City University of New York, 695 Park Ave., New York, NY, 10065, USA; 6 Physics Department, Brooklyn College of the City University of New York, 2900 Bedford Avenue, Brooklyn, NY 11210, USA, Fax: +1 718-951-4407, Tel +1 718-951-5000, ext. 2869

**Keywords:** cyclic phosphonium, ionic liquid, organophosphorus, phosphinanium, phospholanium, phosphonium

## Abstract

Ionic liquids (ILs) incorporating cyclic phosphonium cations are a novel category of materials. We report here on the synthesis and characterization of four new cyclic phosphonium bis(trifluoromethylsulfonyl)amide ILs with aliphatic and aromatic pendant groups. In addition to the syntheses of these novel materials, we report on a comparison of their properties with their ammonium congeners. These exemplars are slightly less conductive and have slightly smaller self-diffusion coefficients than their cyclic ammonium congeners.

## Introduction

The most widely investigated and commercially available ionic liquids (ILs) are composed primarily of nitrogen-centered (particularly imidazolium, pyridinium, pyrrolidinium, and related quaternary ammonium) cations with a wide range of anions [1]. While a number of these ILs have been applied successfully to commercial processes [1], efforts are continually underway to synthesize ILs with improved properties (such as viscosity, thermal stability, melting point, etc.) to expand their applications into larger scale processes in diverse areas. Along that vein, phosphonium ILs have been observed to have favorable characteristics, including lower costs [2–6], lower viscosity (by ~50%) [7–11], greater thermal stability (by ~100 °C) [10–12], and wider electrochemical windows compared to their ammonium congeners. The family of ILs bearing cyclic cations (pyrrolidinium, piperidinium, and azepanium) [13–14] have been shown to have better transport and physical properties than most acyclic tetraalkylammonium salts. While cyclic phosphonium salts have been noted in patent applications [15], their characteristics have not been reported therein nor in journals, even though the comparisons noted above suggest those properties might be advantageous. To address such questions, we report here on the synthesis and physical characterization of some phosphonium analogues to pyrrolidinium and piperidinium ILs, i.e. phospholanium and phosphinanium ILs respectively.

The favorable stability of phosphonium ILs makes them good choices for applications where the IL would be exposed to extreme conditions, such as environmental exposure to high temperatures or exposure to extreme electromagnetic radiation (as in a dye-sensitized solar cell or ionizing radiation as in the recycling of spent nuclear fuel). ILs can play important roles in both of these latter areas [16]. While dialkylimidazolium ILs can be efficient extraction media for nuclear separations [17–19], the imidazolium cation is also a good acceptor of radiolytically produced excess electrons. The resultant radical participates in radiation damage mechanisms that alter the IL's properties [20]. On the other hand, phosphonium ILs, known for their durability and already made in large quantities, can be optimized for extraction of specific species from radioactive spent nuclear fuel. Inclusion of phenyl groups, as in two of the new ILs reported here, can stabilize excess charges (electrons or holes) leading to a more durable extraction system. The question of whether cyclic phosphonium ILs would succeed in solar photoconversion or spent fuel processing depends not only on whether their properties can be tuned to the application, but also whether their reactivity under extreme conditions can be managed.

**Scheme 1:**
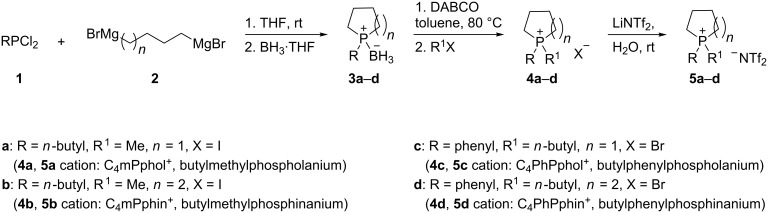
Reaction scheme for the preparation of cyclic phosphonium ionic liquids.

A major challenge for this work has been the handling of the tertiary phosphine precursors, which are pyrophoric and extremely air sensitive. 1-*n*-Butylphospholane and 1-*n*-butylphosphinane were synthesized by the reaction of *n*-butyldichlorophosphine [21] with the appropriate bis-Grignard reagents, BrMg(CH_2_)_4_MgBr and BrMg(CH_2_)_5_MgBr respectively. The resultant cyclic phosphines are air sensitive and therefore were allowed to react with BH_3_·THF to provide the air-stable 1-*n*-butylphospholane–borane and 1-*n*-butylphosphinane–borane complexes that were purified by silica-gel chromatography. The pure compounds were then treated under nitrogen with 1,4-diazabicyclo[2.2.2]octane (DABCO) at 80 °C to remove the borane, and the resultant mixture was purified in a nitrogen-filled glove box on a short silica-gel column. The pure cyclic phosphines (1-*n*-butylphospholane and 1-*n*-butylphosphinane) were allowed to react immediately with iodomethane at room temperature to produce the 1-*n*-butyl-1-methylphospholanium iodide and 1-*n*-butyl-1-methylphosphinanium iodide, respectively. These materials were each mixed with an equivalent amount of lithium bis(trifluoromethylsulfonyl)amide (LiNTf_2_) in water at room temperature to produce the water-insoluble salts, 1-*n*-butyl-1-methylphospholanium bis(trifluoromethylsulfonyl)amide and the corresponding 1-*n*-butyl-1-methylphosphinanium bis(trifluoromethylsulfonyl)amide. In a corresponding manner were prepared the 1-phenylphospholane and the 1-phenylphosphinane by the reaction of dichlorophenylphosphine with the appropriate bis-Grignard reagents, BrMg(CH_2_)_4_MgBr and BrMg(CH_2_)_5_MgBr, respectively. The resulting five-membered cyclic 1-phenylphospholane was isolated in the same manner as was the 1-*n*-butylphospholane: i.e. by conversion with BH_3_·THF to give the air-stable 1-phenylphospholane–borane complex, followed by silica-gel chromatography and then removal of the borane with DABCO, to give the pure 1-phenylphospholane. The six-membered cyclic phosphine, which appears to be less air sensitive than the 1-butylphosphinane species, was purified in open air using a short column of silica gel. Both cyclic phosphines were each allowed to react with 1-bromobutane at reflux under nitrogen to give the 1-*n*-butyl-1-phenylphospholanium bromide (**4c**) and the 1-*n*-butyl-1-phenylphosphinanium bromide (**4d**), respectively. Each of these salts was then mixed with aqueous LiNTf_2_ to form the 1-*n*-butyl-1-phenylphospholanium bis(trifluoromethylsulfonyl)amide (**5c**) and the corresponding 1-*n*-butyl-1-phenylphosphinanium bis(trifluoromethylsulfonyl)amide (**5d**). The overall synthetic route is summarized in Scheme 1.

## Results and Discussion

The conductivities, viscosities, self-diffusion coefficients and thermal properties of the phosphonium ILs **5a–d** and their ammonium congeners are given in Table 1.

**Table 1:** Physical characteristics of cyclic phosphonium NTf_2_ ILs.^a^

Cation^b^	Viscosity cP	Conductivity mS/cm	*D*^+^ (^1^H) 10^−7^ cm^2^/s	*D*^−^ (^19^F) 10^−7^ cm^2^/s	*T*_g_ °C	*T*_m_ °C	*T*_d_ °C

**5a**: C4mPphol^+^	101	2.2 (23 °C)	1.27	1.01	−85	15	444
C4mPyrr^+^	75 [22]	2.8 [22]	2.2 [23] (30 °C)	1.8 [23] (30 °C)	−89	−8 [24]	431 [22]
**5b**: C4mPphin^+^	171	0.92	0.65	0.68	−78	6	386
C4mPip^+^	183 [25]	1.2 [25]	0.98 [25]	0.85 [25]	−77 [26]	−25^c^	400 [25]
**5c**: C4PhPphol^+^	223	–	–	–	−66	29	422
**5d**: C4PhPphin^+^	48 (75 °C)	13.3 (76 °C)	1.56 (75 °C)	2.47 (75 °C)	−48	54	387

^a^All values are for measurements at 25 °C except where noted. Viscosities are calculated from VFT fits. All glass-transition temperatures (*T*_g_) and melting points (*T*_m_) are onset temperatures. Water contents of samples ranged from 76–155 ppm. Uncertainties of viscosities, conductivities, diffusion coefficients (*D^+^* and *D*^−^) are 1 cP, 7%, 5% and 1 °C respectively. *T*_d_ is the thermal decomposition temperature. ^b^Abbreviations and structures of ammonium congeners: NTf_2_ - bis(trifluoromethylsulfonyl)amide anion; C_4_mPphol - butylmethylphospholanium; C_4_mPyrr - butylmethylpyrrolidinium; C_4_mPphin - butylmethylphosphinanium; C_4_mPip - butylmethylpiperidinium; C_4_PhPphol - butylphenylphospholanium; C_4_PhPphin - butylphenylphosphinanium.
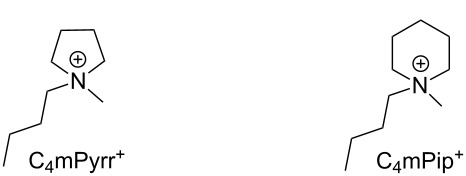
^c^Value subject to dispute, either −25 °C [25] or just below 0 °C [27].

Comparison of the five-membered cyclic cation ILs C_4_mPphol NTf_2_ **5a** and C_4_mPyrr NTf_2_ (butylmethylpyrrolidinium NTf_2_) reveals that the viscosity of the phosphonium salt is one third higher than that of the ammonium salt (101 cP vs 75 cP at 25 °C). For the six-membered rings the phosphonium IL C_4_mPphin NTf_2_ **5b** is slightly less viscous than C_4_mPip NTf_2_ (butylmethylpiperidinium NTf_2_), 171 cP vs 183 cP. Interestingly, the cyclic cations do not follow the common trend among the quaternary tetra-*n*-alkylated cations wherein the room temperature viscosities of the phosphonium salts are approximately one-half of those of their ammonium congeners [7–11]. This difference draws attention to the particular role that the cyclic alkyl functionality plays in IL fluid dynamics in contrast to two linear chains. This topic merits further exploration via molecular dynamics simulations [28] and physical studies such as NMR diffusion measurements, nuclear Overhauser effect spectroscopies, and quasi-elastic neutron scattering. As seen for instance for the cyclic ammonium salts, the viscosities of the cyclic phosphonium salts increase with increasing ring size for two of the compounds synthesized. As suggested by the viscosity results, the conductivities of the phosphonium ILs are lower than those for their ammonium congeners, and that for both families they decrease with increasing ring size. The conductivity of C_4_mPphol NTf_2_ (2.2 mS/cm) is slightly smaller than that of the C_4_mPyrr (2.8 mS/cm) although not as much as expected from the viscosity difference. The self-diffusion coefficients of the anion (*D*^−^) and the cation (*D^+^*) are somewhat lower for the phosphonium salts than the ammonium ones. Interestingly, despite its slightly lower viscosity, C_4_mPphin IL has lower diffusion coefficients than C_4_mPip NTf_2_.

For the P-versus-N pairs where direct comparisons can be made, the glass-transition temperatures *T*_g_ are very close, allowing for experimental differences. In contradiction to the trend for linear tetraalkyl cations, the melting temperatures (*T*_m_) for the cyclic phosphonium salts are higher than those of their cyclic ammonium congeners. C_4_mPphol NTf_2_ melts 23 °C higher than does C_4_mPyrr NTf_2_, and C_4_mPphin NTf_2_ melts 31 °C higher than does C_4_mPip NTf_2_. Henderson and Passerini [29] showed that the often-cited *T*_m_ of −18 °C for C_4_mPyrr NTf_2_ was due to a metastable crystalline form by using an annealing process. The DSC scan of un-annealed C_4_mPphol NTf_2_ shows all the thermal features exhibited by un-annealed C_4_mPyrr NTf_2_ (glass transition, cold crystallization, solid–solid transition, and melting). When the recommended annealing method [24] was applied to C_4_mPphol NTf_2_ the first three thermal features listed just above were eliminated, but the position of the melting point did not change. The apparent thermal decomposition temperature (*T*_d_) of the cyclic phosphonium NTf_2_ ILs are not particularly different from those of the cyclic ammonium NTf_2_ ILs, which is similar to the situation of linear alkyl quaternary cation ILs [11]. In some instances where the anion is nucleophilic, such as with dicyanamide [12], or the leaving group is resonance-stabilized, such as a benzyl radical [10], the phosphonium cations have greater thermal stability than the ammonium ones. One trend observed for both cation types is that the ILs with five-membered rings have greater thermal stability than those with six-membered rings. Further work will be required to determine if the observed TGA behaviors are due to actual decomposition or to volatility of the ILs [30].

While developing our synthetic approach to the aliphatic C_4_mPphol^+^ and C_4_mPphin^+^ cations, which requires the use of highly air-sensitive reagents and intermediates as described above, we refined our techniques using less reactive phenylphosphines. The resulting butylphenylphospholanium (C_4_PhPphol NTf_2_) and butylphenylphosphinanium (C_4_PhPphin NTf_2_) ionic liquids are examples of a rare class of ILs with direct attachment of the aryl group to the cationic center. We were unable to find any information on ILs containing analogous nitrogen-centered cations for comparison, although ILs with aryl groups attached directly to the nitrogen atoms of imidazolium cations are reported [31]. The melting points of the phenylphosphonium ILs are both above room temperature (see Table 1), however C_4_PhPphol NTf_2_ is persistently metastable as a supercooled liquid under ambient conditions. The most significant effects observed for the replacement of a methyl group with a phenyl ring in this family of cations are the substantial increases of 19 °C and 30 °C in the glass-transition temperatures for the 5- and 6-membered ring cations respectively. Similar effects on *T*_g_ have been reported for other classes of ILs bearing benzyl groups [32]. This family of cations may have the useful attributes for reducing radiation-induced damage of the IL owing to the stabilization of excess charges on the phenyl groups.

## Conclusion

We have successfully prepared a series of new cyclic phosphonium ILs that are representatives of a wide class of potentially useful ILs. These exemplars are slightly less conductive and have slightly smaller self-diffusion coefficients than their cyclic ammonium congeners. Their viscosities are also lower than their ammonium congeners. In other instances, such as the peculiarities in melting point and viscosity trends of the C-2-methylated imidazolium salts [33], ether-substituted ILs [34], and bis(oxalato)borate salts [35], detailed investigation into those intriguing behaviors led to deeper insights into the dynamical workings of ILs that have had important impacts on the field. For this reason we will continue to explore cyclic phosphonium ILs through synthesis of a wider variety of structural types and substituents, variation of anions, and more extensive characterization of their physical, structural, and dynamical properties. In particular, this family of ILs, by comparison with the more familiar cyclic ammonium ILs, should reveal the dynamical effects of cyclic vs linear alkyl chains on the physical properties of ionic liquids.

## Supporting Information

Detailed synthesis and characterization procedures are provided for all compounds synthesized and characterized. NMR spectra are provided for all compounds for which NMR data is reported. DSC thermograms are provided for the bis(trifluoromethylsulfonyl)amide ionic liquids (**5a**–**5d)**.

File 1 NMR Spectra.

File 2 DSC Thermograms.

File 3 Experimental.
