# Supplementary material for: Cyclic phosphonium ionic liquids
Source: Beilstein J Org Chem. 2014 Jan 24;10:271–5. doi: 10.3762/bjoc.10.22 (PMC3944138; doi:10.3762/bjoc.10.22)

Supporting Information
for
Cyclic phosphonium ionic liquids

Sharon I. Lall-Ramnarine*[1], Joshua A. Mukhlall[1,2], James F. Wishart[*3], Robert R. Engel[*2], Alicia R. Romeo[1], Masao Gohdo[3,4], Sharon Ramati[1,2], Marc Berman[5], and Sophia N. Suarez[6]

Address: [1]Chemistry Department, Queensborough Community College of the City University of New York, 222-05 56th Avenue, Bayside, NY, 11364, USA, Fax: +1 718-281-5078, Tel: +1 718-281-5572, [2]Department of Chemistry and Biochemistry, Queens College of the City University of New York, 65-30 Kissena Boulevard, Flushing, NY, 11367, USA, Fax: +1 718-997-5531, Tel: +1 718-997-4106, [3]Chemistry Department, Brookhaven National Laboratory, Upton, NY, 11973, USA, Fax: +1 631-344-5815, Tel: +1 631-344-4327, [4]The Institute of Scientific and Industrial Research (ISIR), Osaka University, 8-1 Mihogaoka, Ibaraki, Osaka 567-0047, Japan, [5]Department of Physics and Astronomy, Hunter College of the City University of New York, 695 Park Ave., New York, NY, 10065, USA and [6]Physics Department, Brooklyn College of the City University of New York, 2900 Bedford Avenue, Brooklyn, NY 11210, USA, Fax: +1 718-951-4407, Tel +1 718-951-5000, ext. 2869

Email: Sharon Lall-Ramnarine - slallramnarine@qcc.cuny.edu; James F. Wishart - wishart@bnl.gov; Robert R. Engel - Robert.engel@qc.cuny.edu

*Corresponding author

DSC Thermograms

Butylmethylphospholanium NTf_2 (5a)

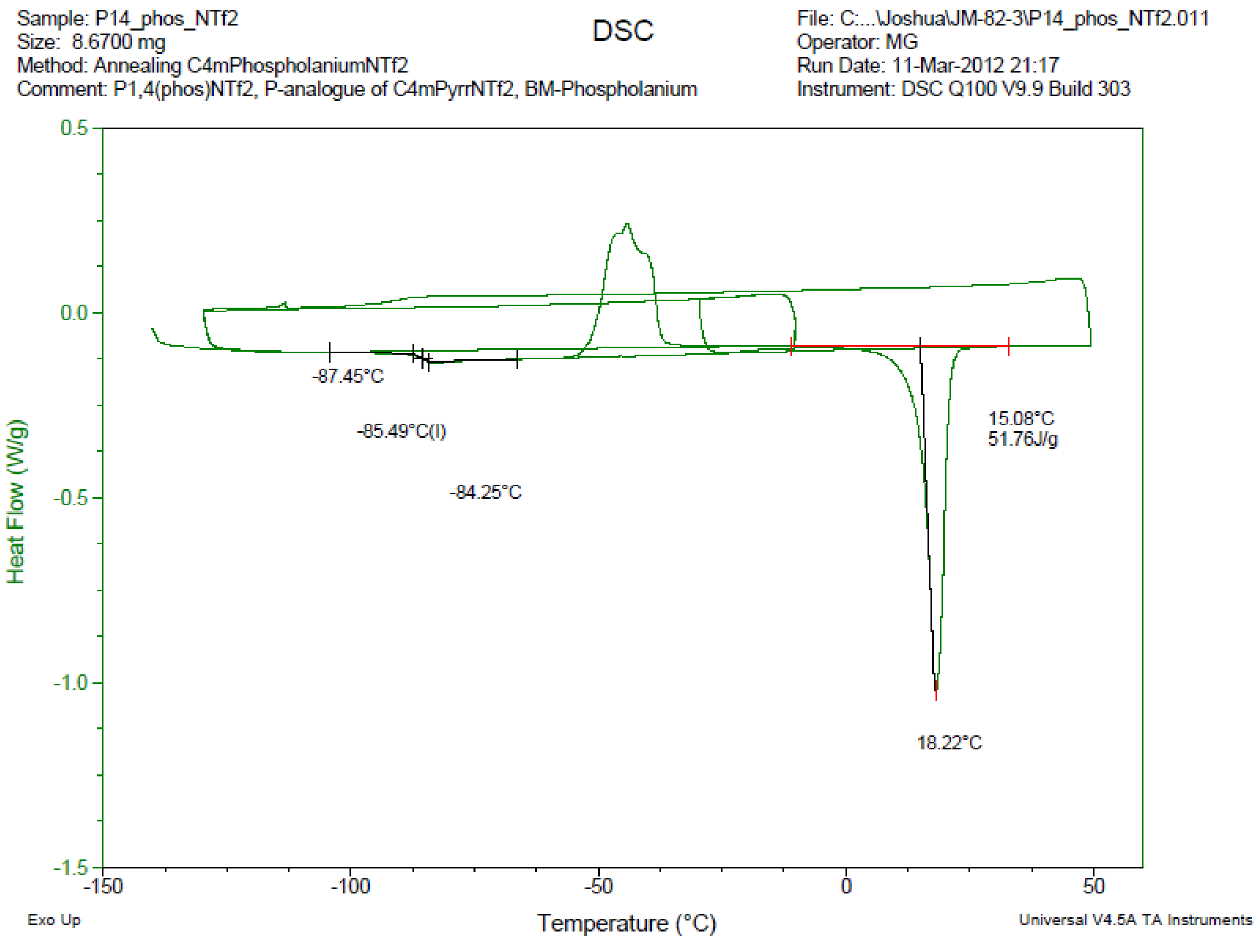

Butylmethylphosphinanium NTf_2 (5b)

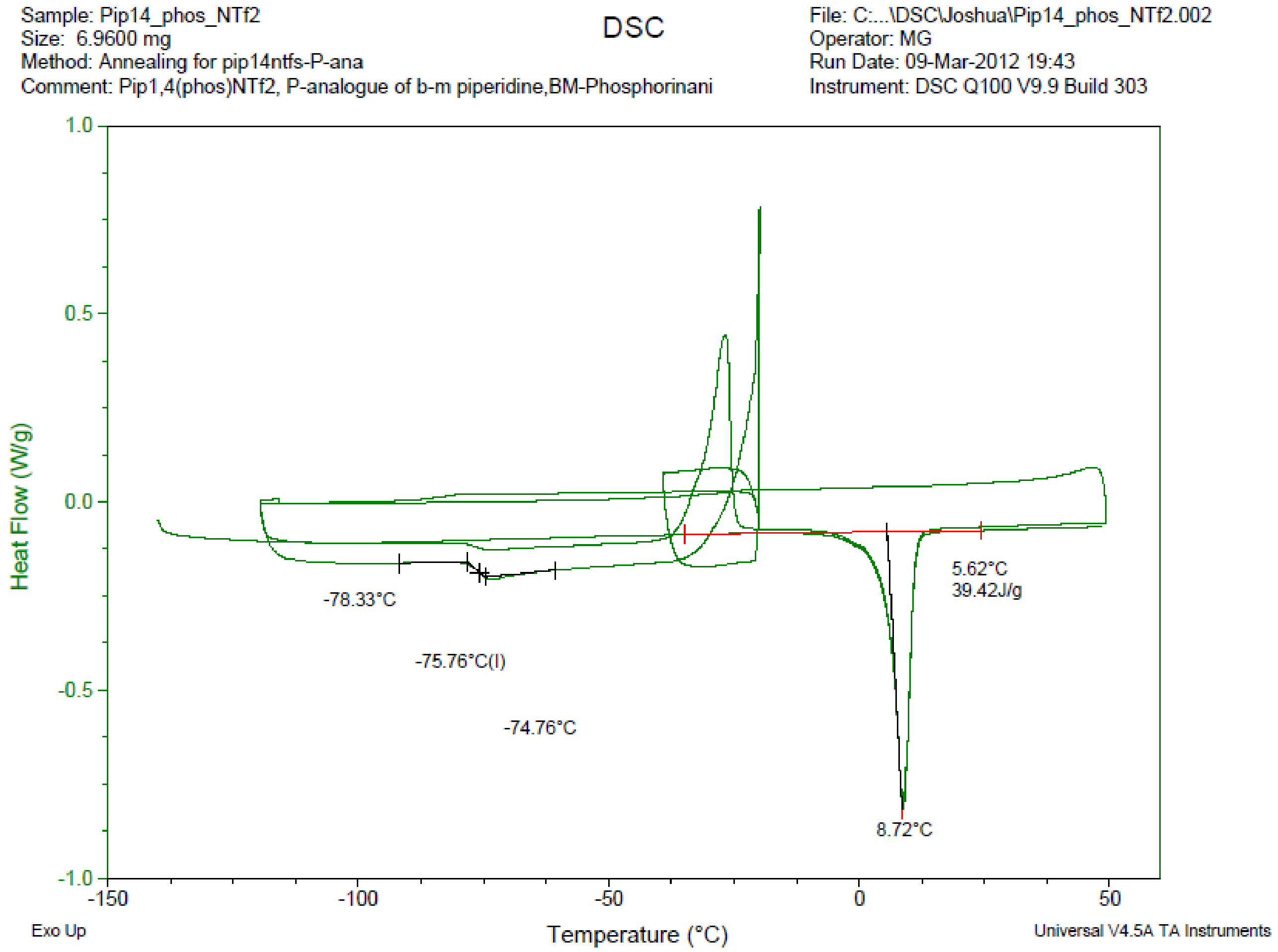

Butylphenylphospholanium NTf_2 (5c)

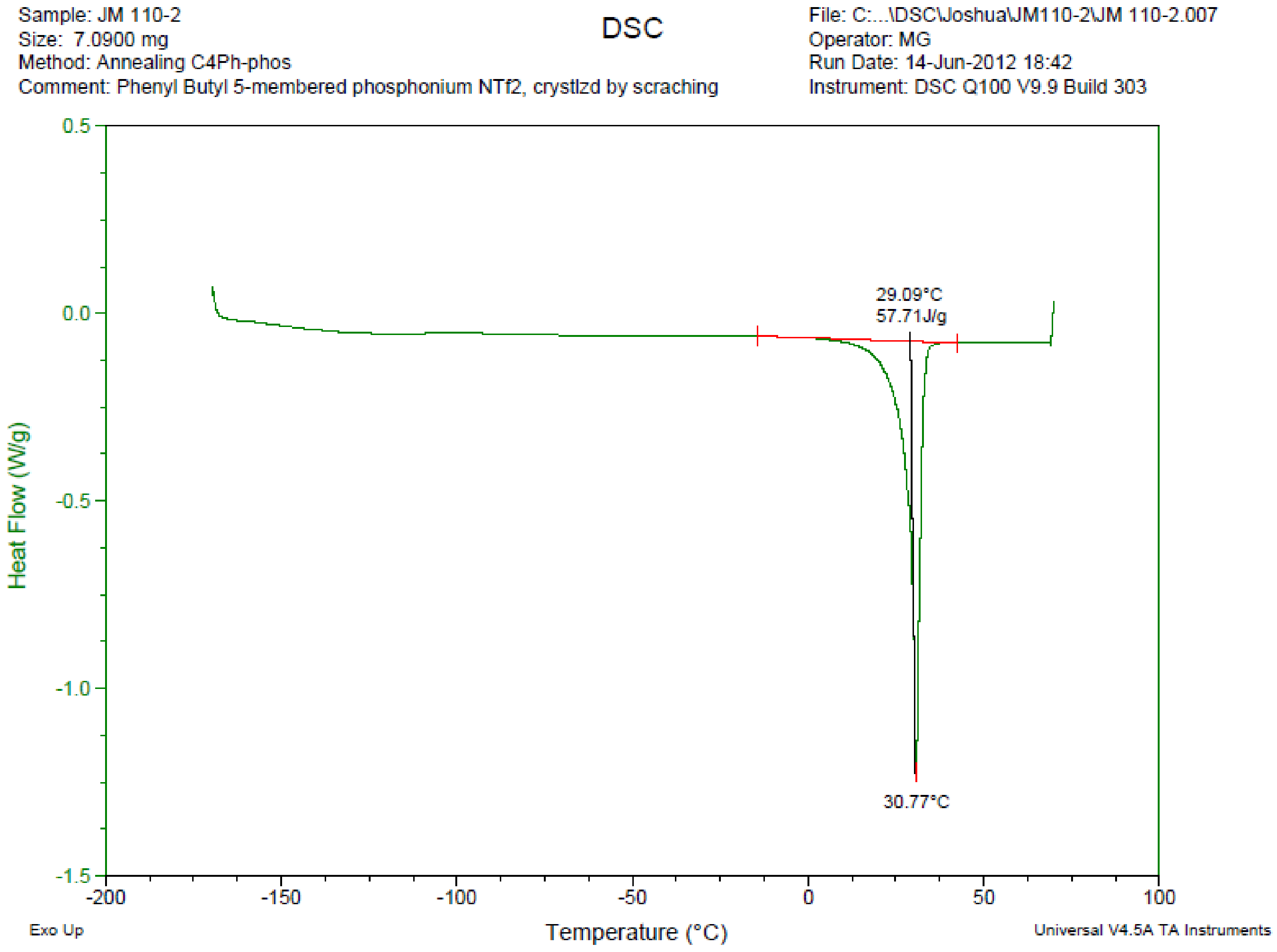

Butylphenylphospholanium NTf_2 (5c)

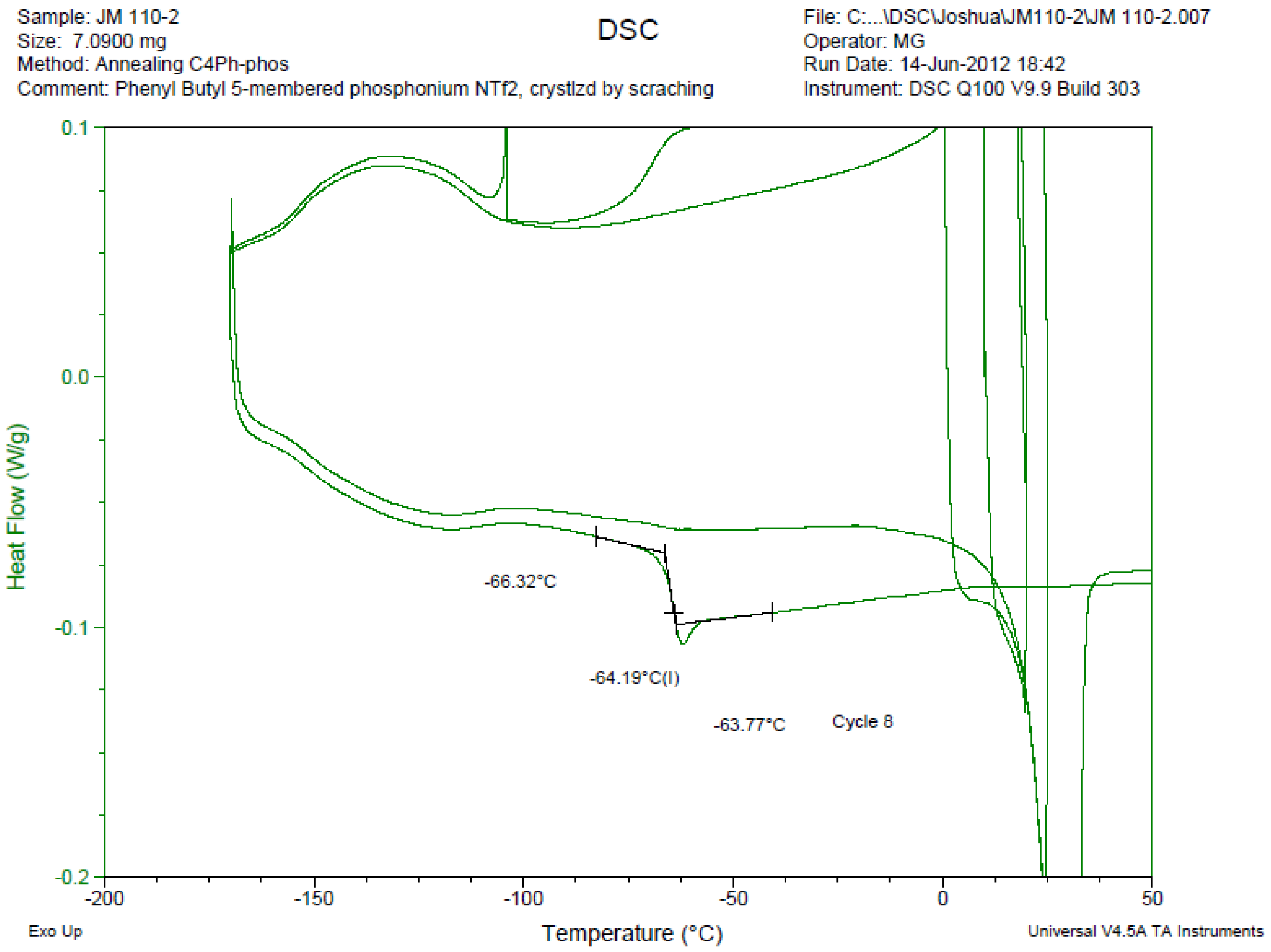

Butylphenylphosphinanium NTf_2 (5d)

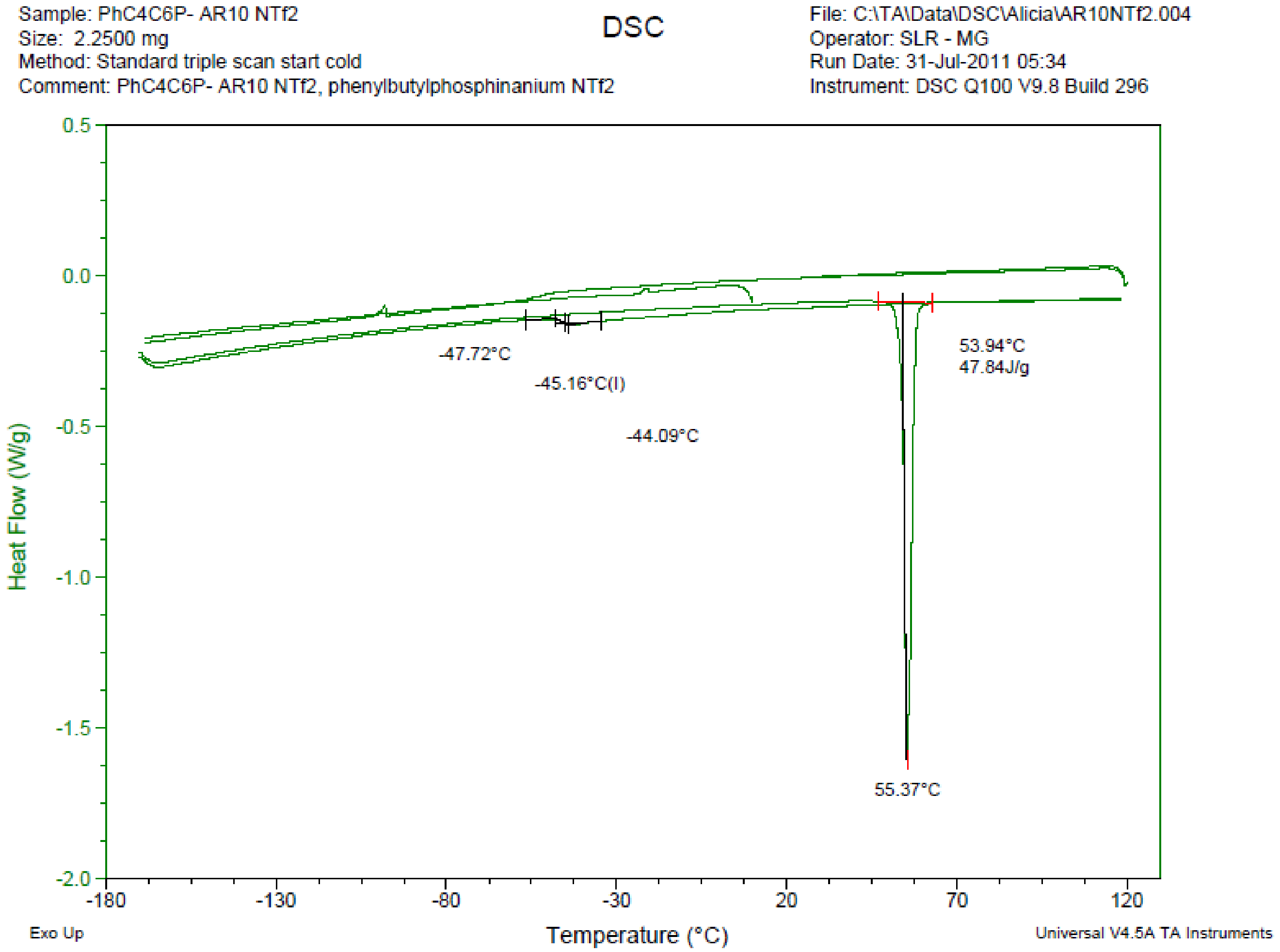

Supplement: File 2 — DSC Thermograms. [file Beilstein_J_Org_Chem-10-271-s002.pdf]
