# Supplementary material for: Cyclic phosphonium ionic liquids
Source: Beilstein J Org Chem. 2014 Jan 24;10:271–5. doi: 10.3762/bjoc.10.22 (PMC3944138; doi:10.3762/bjoc.10.22)
Supplement: File 3 — Experimental. [file Beilstein_J_Org_Chem-10-271-s003.pdf]

Supporting Information
for
Cyclic phosphonium ionic liquids

Sharon I. Lall-Ramnarine*[1], Joshua A. Mukhlall[1,2], James F. Wishart[*3], Robert R. Engel[*2], Alicia R. Romeo[1], Masao Gohdo[3,4], Sharon Ramati[1,2], Marc Berman[5], and Sophia N. Suarez[6]

Address: [1]Chemistry Department, Queensborough Community College of the City University of New York, 222-05 56th Avenue, Bayside, NY, 11364, USA, Fax: +1 718-281-5078, Tel: +1 718-281-5572, [2]Department of Chemistry and Biochemistry, Queens College of the City University of New York, 65-30 Kissena Boulevard, Flushing, NY, 11367, USA, Fax: +1 718-997-5531, Tel: +1 718-997-4106, [3]Chemistry Department, Brookhaven National Laboratory, Upton, NY, 11973, USA, Fax: +1 631-344-5815, Tel: +1 631-344-4327, [4]The Institute of Scientific and Industrial Research (ISIR), Osaka University, 8-1 Mihogaoka, Ibaraki, Osaka 567-0047, Japan, [5]Department of Physics and Astronomy, Hunter College of the City University of New York, 695 Park Ave., New York, NY, 10065, USA and [6]Physics Department, Brooklyn College of the City University of New York, 2900 Bedford Avenue, Brooklyn, NY 11210, USA, Fax: +1 718-951-4407, Tel +1 718-951-5000, ext. 2869

Email: Sharon Lall-Ramnarine - slallramnarine@qcc.cuny.edu; James F. Wishart - wishart@bnl.gov; Robert R. Engel - Robert.engel@qc.cuny.edu

*Corresponding author

Experimental

Materials and methods - All chemicals used for syntheses were of reagent grade and were obtained from the following sources: Sigma–Aldrich (benzophenone, 1-bromobutane, 1,4-dibromobutane, methyl iodide, BH_3·THF, hexane, PCl_3); Pharmco–Aper (toluene, THF, chloroform); Fisher Scientific (acetonitrile, Mg turnings, ethyl acetate); Alfa Aesar (1,5-dibromopentane, dichlorophenylphosphine, DABCO, $LiNTf_2$). Toluene and THF were distilled from sodium benzophenone under nitrogen. Acetonitrile was distilled from calcium hydride under nitrogen. 1-Bromobutane, 1,4-dibromobutane, 1,5-dibromo-pentane, and methyl iodide were degassed and stored over molecular sieves in a nitrogen-filled glove box. Other reagents were used without further purification. Flash-column chromatography was performed on 230–400 mesh silica gel, and TLC was performed on aluminum-backed silica-gel plates (200 µm). ^{1}H, ^{13}C, and ^{31}P NMR spectra were recorded on a 400 MHz Bruker NMR spectrometer with tetramethylsilane and 85% phosphoric acid in deuteriochloroform and D_2O as external standards. Elemental analyses were performed by ALS Environmental (Columbia Analytical Services) of Dallas, TX, USA.

1-n-Butylphospholane–borane (**3a**) - The bis-Grignard reagent $BrMg(CH_2)_4MgBr$ was synthesized by adding 1,4-dibromobutane (13.6 g, 62.9 mmol) dissolved in THF (55 mL) dropwise to magnesium turnings (8.35 g, 343 mmol) in THF (15 mL) over 4 h under argon atmosphere. *n*-Butyldichlorophosphine was synthesized on a 10 g scale according to a literature procedure [1] with the following modifications: (1) the reaction was performed in THF instead of ether;

(2) after the reaction was completed, no purification by distillation was done, but the reaction mixture was filtered via standard Schlenk-type techniques and the solvent removed by evaporation under vacuum to afford the crude product, which was used immediately as follows. Under argon, the crude *n*-butyldichlorophosphine in THF (40 mL) was added dropwise over 30 min to the cold solution (5 °C) of $BrMg(CH_2)_4MgBr$. The reaction mixture was stirred at room temperature for 15 h, after which BH_3·THF solution (70 mL, 70 mmol) was added at 0 °C. The solution was stirred for an additional 1 h, then added slowly with stirring to a mixture of ice (200 g) and 1 M HCl (100 mL). The organic layer was removed and the aqueous layer was washed with ethyl acetate (3 x 80 mL). The combined organic layers were washed with saturated aqueous NaCl solution (100 mL) and then dried with $MgSO_4$. The solvent was removed by rotary evaporation to give a viscous liquid. The crude product was purified by silica-gel chromatography using 4% EtOAc/hexane (for every 2 g of crude liquid, 80 g of silica gel was used in a 40 mm diameter column). All fractions corresponding to R_f 0.42 were combined and then the solvent was evaporated under vacuum to afford compound **3a** as a colorless liquid (2.6 g, 26% yield based on 1,4-dibromobutane used). ^{1}H NMR (400 MHz, $CDCl_3$): 1.35-1.94 (m, 14H), 0.92 (t, J = 7.2 Hz, CH_3, 3H), 0.53 (q, J = 95.6 Hz, BH_3, 3H). ^{13}C NMR (100 MHz, $CDCl_3$): 26.97, 25.69 (d, J = 5.6 Hz) 25.53 (d, J = 22.8 Hz), 24.23 (d, J = 13.1 Hz), 24.12 (d, J = 35.5 Hz), 13.66. ^{31}P (161 MHz, $CDCl_3$): 30.64 (q, J= 55.7 Hz).

1-n-Butylphosphinane–borane (**3b**) - Compound **3b** was synthesized in an analogous manner as **3a** using 1,5-dibromopentane for the preparation of the bis-Grignard reagent $BrMg(CH_2)_5MgBr$. Compound **3b** was isolated as a colorless liquid (3.10 g, 19% yield based on 1,5-dibromopentane used), R_f 0.5 (7% hexane/ethyl acetate on silica gel). ^{1}H NMR (400 MHz, $CDCl_3$): 1.35-1.94 (m, 16H), 0.92 (t, J = 7.2 Hz, CH_3, 3H), 0.43 (q, J = 92.2 Hz, BH_3, 3H). ^{13}C NMR (100 MHz, $CDCl_3$): 26.65 (d, J = 5.2 Hz), 24.40, 24.34 (d, J = 13.2 Hz), 23.02 (d, J = 34.2 Hz), 21.62 (d, J = 41.8 Hz), 21.42 (d, J = 13.2 Hz), 13.64. ^{31}P (161 MHz, $CDCl_3$): 3.16 (q, J= 60.0 Hz).

1-Phenylphospholane–borane (**3c**) - The bis-Grignard reagent $BrMg(CH_2)_4MgBr$ was synthesized by adding 1,4-dibromobutane (15.8 g, 73.0 mmol) dissolved in THF dropwise to magnesium turnings (7.10 g, 290 mmol) in 15 mL of THF over 4 h under argon. After stirring the solution at room temperature for 1 h, dichlorophenylphosphine (14.4 g, 81 mmol) in 80 mL of THF was added dropwise over 30 min to a cold solution (5 °C) of $BrMg(CH_2)_4MgBr$. The reaction solution was stirred at room temperature for 17 h, and afterwards BH_3·THF solution (110 mL, 110 mmol) was added at 0 °C. The solution was stirred for an additional 1 h then added slowly with stirring to a mixture of ice (200 g) and 1 M HCl (100 mL). The organic layer was removed and the aqueous layer was washed with 3 x 100 mL EtOAc. The combined organic layers were washed with saturated NaCl solution (100 mL) and then dried over $MgSO_4$. The solvent was removed by rotary evaporation to give a viscous liquid. The crude product was

purified by silica-gel chromatography using 10% EtOAc/hexane (for every 2 g of crude liquid, 80 g of silica gel were used in a 40 mm diameter column). All fractions corresponding to R_f 0.5 were combined and the solvent evaporated under vacuum to afford the compound **3c** as a colorless liquid (5.8 g, 45% yield based on 1,4-dibromobutane used). ^{1}H NMR (400 MHz, $CDCl_3$): 7.68-7.74, (m, 2H), 7.41-7.51 (m, 3H), 1.97-2.25 (m, 8H), 0.85 (br q, J = 95.3 Hz, BH_3 3H). ^{13}C NMR (100 MHz, $CDCl_3$):131.58, 131.46 (d, J = 9.0 Hz), 131.13 (d, J = 2.3 Hz), 128.86 (d, J = 9.8 Hz), 27.62, 26.93 (d, J = 37.1 Hz). ^{31}P (161 MHz, $CDCl_3$): 28.85 (q, J= 53.6 Hz).

1-n-Butyl-1-methylphospholanium iodide (**4a**) - In a nitrogen-filled glove box, 1-*n*-butylphospholane–borane (**3a**) (1.55 g, 9.81 mmol) and DABCO (6.83 g, 60.9 mmol) were dissolved in toluene (130 mL). The flask was sealed and the solution heated at 80 °C while stirring for 24 h. The toluene was evaporated under vacuum to give a mixture of a white solid and a viscous liquid. The mixture was purified in the glove box by passing it through a short column of silica gel with THF and all material (the cyclic phosphine) that moved with the solvent front was collected. To this solution iodomethane (2.5 mL, 40 mmol) was added immediately and the solution was stirred under nitrogen at room temperature overnight. The 1-*n*-butyl-1-methylphospholanium iodide (**4a**) precipitated as a white solid. The solvent was decanted and the product dried under vacuum to give the desired material (2.4 g, 86% yield). ^{1}H NMR (400 MHz, D_2O): 1.57-1.76 (m. 6H), 1.37-1.47 (m, 4H), 1.29 (d, J = 14.4 Hz, CH_3, 3H), 0.81-1.04 (m, 4H),

0.31 (t, J = 7.3 Hz, CH_3 3H).^{13}C NMR (100 MHz, D_2O): 25.61 (d, J = 5.5 Hz), 23.43 (d, J = 4.6 Hz), 23.16 (d, J = 16.0 Hz), 21.66 (d, J = 52.5 Hz), 21.45 (d, J = 46.0 Hz), 12.62, 6.21 (d, J = 49.0 Hz). ^{31}P (161 MHz, D_2O): 51.38.

1-n-Butyl-1-methylphosphinanium iodide (**4b**) - This compound was synthesized in the same manner as **4a**, using **3b** to give **4b** as a white solid (1.06 g, 85% yield). ^{1}H NMR (400 MHz, $CDCl_3$): 2.44-2.69 (m, 6H), 2.18 (d, J = 13.7 Hz, CH_3, 3H), 1.48-2.12 (m, 10H), 0.97 (t, J = 7.0 Hz, CH_3 3H).^{13}C NMR (100 MHz, $CDCl_3$): 24.44 (d, J = 7.6 Hz), 23.79 (d, J = 15.5 Hz), 23.30(d, J = 4.5 Hz), 21.04 (d, J = 6.7 Hz), 21.67 (d, J = 49.1 Hz), 18.92 (d, J = 48.1 Hz), 13.63, 5.54 (d, J = 51.6 Hz). ^{31}P (161 MHz, $CDCl_3$): 22.31.

1-n-Butyl-1-phenylphospholanium bromide (**4c**) - The deprotection of the cyclic phosphine was done in 1 g portions as follows: in a nitrogen-filled glove box, 1-phenylphospholane–borane (**3c**) (1.00 g, 5.62 mmol) and DABCO (3.20 g, 28.5 mmol) were dissolved in toluene (30.0 mL). The flask was sealed and the solution heated at 85 °C with stirring for 24 h. Toluene was evaporated under vacuum to give a mixture of a white solid and a viscous liquid. The mixture was purified in the glove box by passing it through a short column of silica gel (~50 g) with THF and all material (the cyclic phosphine) that moved with the solvent front was collected. The solvent was evaporated under reduced pressure and the pure phosphine was dissolved in 30 mL acetonitrile under nitrogen. Then 1-bromobutane (0.92 g, 6.71 mmol) was added to the solution. The reaction

mixture was refluxed under nitrogen for 2.5 h and then rotary-evaporated to afford 1-*n*-butyl-1-phenylphospholanium bromide (**4c**) as a clear slightly yellow liquid (1.50 g, 89%). ^{1}H NMR (400 MHz, $CDCl_3$): δ 7.94–8.00 (m, 2H), 7.60–7.72 (m, 3H), 3.01–3.15 (m, 4H), 2.57–2.68 (m, 2H), 2.29–2.46 (m, 2H), 2.06 (m, 2H), 1.41–1.51 (m, 4H), 0.85 (t, J = 6.9 Hz, CH_3, 3H). ^{31}P NMR (161 MHz, $CDCl_3$): δ 50.61.

1-n-Butyl-1-phenylphosphinanium bromide (**4d**) - Under a nitrogen atmosphere, dichlorophenylphosphine (12.1 g, 67.6 mmol) in THF (40 mL) was added dropwise over 30 min to a cold solution (5 °C) of bis-Grignard reagent $BrMg(CH_2)_5MgBr$ (**2b**) prepared from Mg turnings (7.07 g, 291 mmol) and 1,5-dibromopentane (15.5 g, 67.6 mmol) in THF (130 mL). After stirring the reaction solution for 4 h, it was filtered and the solvent evaporated under vacuum to get a mixture of a liquid and a solid. The mixture was washed with 5 x 100 mL hexane and the hexane extracts combined. The solvent was evaporated under vacuum to give a gum which was passed through a short column of silica gel with the aid of CH_2Cl_2/hexane 3:1. All material moving with the solvent front was collected and the solvent evaporated under vacuum to give the 1-phenylphosphinane (4.2 g) contaminated with another phosphorus-containing impurity. The material was not purified further but was converted immediately to 1-*n*-butyl-1-phenylphosphinanium bromide (**4d**) as follows: under nitrogen the crude cyclic phosphine and 1-bromobutane (4.84 g, 35.3 mmol) were heated to reflux in acetonitrile (60 mL) for 2 h. The solvent was evaporated under vacuum to give a

mixture of gum and a solid which was recrystallized from a mixture of acetonitrile and diethyl ether to give 1-*n*-butylphosphinanium bromide (**4d**) as a white solid (4.2 g, 20.0% based on phenyldichlorophosphine used). ^{1}H NMR (400 MHz, D_2O): 7.06-7.26 (m. 5H, Ar*H*), 2.04-2.14 (m, 2H), 1.75-1.94 (m, 4H), 1.34-1.49 (m. 2H), 0.98-1.22 (m. 4H), 0.66-0.82 (m, 4H), 0.18 (t, *J* = 7.1 Hz, CH_3 3H).^{13}C NMR (100 MHz, D_2O): 134.45 (d, *J* = 2.9 Hz), 131.63 (d, *J* = 9.2 Hz), 130.08 (d, *J* = 11.9 Hz), 117.50 (d, *J* = 48.7 Hz), 20.48 (d, *J* = 6.3 Hz), 17.07, 6.21 (d, *J* = 48.4 Hz), 13.75. ^{31}P (161 MHz, D_2O): 20.01.

1-n-Butyl-1-methylphospholanium bis(trifluoromethylsulfonyl)amide (**5a**) - $LiNTf_2$ (3.50 g, 12.2 mmol) dissolved in distilled water (10 mL) was added to 1-*n*-butyl-1-methylphospholanium iodide (**4a**) (2.90 g, 10.14 mmol) dissolved in distilled water (40 mL). The solution was stirred for 12 h after which the solvent was evaporated on a rotary evaporator and the resultant liquid was dissolved in chloroform (50 mL) and washed with distilled water until no halide was detected (the washings were tested with 50 mM aqueous $AgNO_3$). The solvent was then evaporated and the liquid dried under high vacuum at 35 °C for 24 h to remove residual water. The resultant 1-*n*-butyl-1-methylphospolanium bis(trifluoromethyl-sulfonyl)amide was isolated as a clear, colorless liquid. (4.20 g, 94% yield). ^{1}H NMR (400 MHz, $CDCl_3$): 2.20-2.35 (m, 6H), 1.99-2.16 (m, 4H), 1.92 (d, *J* = 14.1 Hz, CH_3, 3H), 1.45-1.58 (m, 4H), 0.96 (t, *J* = 7.0 Hz, CH_3 3H). ^{13}C NMR (100 MHz, $CDCl_3$): 119.81 (q, *J* = 321.7 Hz), 25.89 (d, *J* = 5.6 Hz), 24.00 (d, *J* = 4.9 Hz), 23.54 (d, *J* = 16.1 Hz), 22.07 (d, *J* = 51.8 Hz), 21.9 (d, *J* = 45.4 Hz), 13.22,

6.91 (d, J = 48.4 Hz). ^{31}P (161 MHz, $CDCl_3$): 51.75. Calculated for $C_{11}H_{20}NO_4S_2PF_6$: C, 30.07%; H, 4.59%; N, 2.84%. Found: C, 30.39%; H, 4.92%; N, 3.07%.

1-n-Butyl-1-methylphosphinanium bis(trifluoromethylsulfonyl)amide (**5b**) - $LiNTf_2$ (1.22 g, 4.25 mmol) dissolved in distilled water (5 mL) was added to 1-*n*-butyl-1-methylphosphinanium iodide (**4b**) (1.06 g, 3.53 mmol) dissolved in distilled water (30 mL). The solution was stirred for 12 h, after which the hydrophobic ionic liquid was extracted with 3 x 100 mL chloroform. The solvent was evaporated on a rotary evaporator and the resultant liquid was dissolved in chloroform (25 mL) and washed with distilled water until no halide was detected (the washings were tested with 50 mM aqueous $AgNO_3$). The solvent was evaporated and the liquid dried under high vacuum at 35 °C for 24 h to remove residual water. The resultant 1-*n*-butyl-1-methylphosphinanium bis(trifluoromethylsulfonyl)amide was isolated as a clear colorless liquid (1.50 g, 94% yield). ^{1}H NMR (400 MHz, $CDCl_3$): 2.12-2.28 (m, 6H), 1.46-2.06 (m, 10H), 1.84 (d, J = 13.6 Hz, CH_3, 3H), 0.95 (t, J = 7.0 Hz, CH_3 3H).^{13}C NMR (100 MHz, $CDCl_3$): 119.82 (q, J = 321.7 Hz), 24.47 (d, J = 7.6 Hz), 23.60 (d, J = 16.2 Hz), 22.96 (d, J = 4.9 Hz), 20.71 (d, J = 6.4 Hz), 20.35 (d, J = 49.1 Hz), 18.38 (d, J =48.1 Hz), 13.21, 3.99 (d, J = 52.2 Hz). ^{31}P (161 MHz, $CDCl_3$): 22.08. Calculated for $C_{12}H_{22}NO_4S_2PF_6$: C, 31.79%; H, 4.89%; N, 3.09%. Found: C, 32.14%; H, 5.01%; N, 3.18%.

1-n-Butyl-1-phenylphospholanium bis(trifluoromethylsulfonyl)amide (**5c**) - $LiNTf_2$ (3.48 g, 12.1 mmol) dissolved in distilled water (10 mL) was added to 1-*n*-butyl-1-phenylphospholanium bromide (**4c**) (3.04 g, 10.1 mmol) dissolved in distilled water (50 mL) at room temperature. The solution was stirred for 12 h after which the hydrophobic ionic liquid was extracted with 3 x 50 mL chloroform. The solvent was removed by rotary evaporation and the resultant liquid was dissolved in chloroform (50 mL) and washed with distilled water until no halide was detected (the washings were tested with 50 mM aqueous $AgNO_3$). The solvent was evaporated and the resultant liquid was dried under high vacuum at 35 °C for 24 h to remove residual water. The resultant 1-*n*-butyl-1-phenylphospholanium bis(trifluoromethylsulfonyl)amide was isolated as a colorless liquid (4.6 g, 91% yield). On standing, the supercooled liquid produced a white solid. ^1H NMR (400 MHz, $CDCl_3$): 7.66-7.80 (m, 5H), 2.48-2.72 (m, 6H), 2.06-2.31 (m, 4H), 1.40-1.52 (m, 4H), 0.89 (t, J = 7.0 Hz, CH_3 3H). ^{13}C NMR (100 MHz, $CDCl_3$): 134.76 (d, J = 2.9 Hz), 131.54 (d, J = 9.5 Hz), 130.47 (d, J = 12.1 Hz) 119.90 (q, J = 321.4 Hz), 130.47 (d, J = 12.1 Hz), 26.14 (d, J = 5.6 Hz), 24.29 (d, J = 5.1 Hz), 23.45 (d, J = 16.1 Hz), 22.33 (d, J = 51.9 Hz), 22.75 (d, J = 44.8 Hz), 13.20. ^{31}P (161 MHz, $CDCl_3$): 50.21. Calculated for $C_{16}H_{22}NO_4S_2PF_6$: C, 38.32%; H, 4.42%; N, 2.79%. Found: C, 38.87%; H, 4.97%; N, 3.27%.

1-n-Butyl-1-phenylphosphinanium bis(trifluoromethylsulfonyl)amide (**5d**) - $LiNTf_2$ (3.82 g, 13.3 mmol) dissolved in 95% ethanol (20 mL) was added to 1-*n*-butyl-1-phenylphosphinanium bromide (**4d**) (4.20 g, 13.3 mmol) dissolved in 95%

ethanol (20 mL) at room temperature. The solution was stirred for 24 h and then submitted to rotary evaporation to give 1-*n*-butyl-1-phenylphosphinanium bis(trifluoromethylsulfonyl)amide as a white solid. Removal of LiBr from the solid was performed by stirring the solid with deionized water (10 mL) for 20 min at 65 °C, followed by cooling the mixture in an ice bath and extraction with water using a pipet. This procedure was repeated with portions (10 mL) of deionized water until no halide salt was detected (the water extracts were tested with 50 mM aqueous $AgNO_3$). The product was dried under high vacuum at 60 °C for 2 d to remove any residual water (4.0 g, 59% yield). The resultant sample was a white solid. ^{1}H NMR (400 MHz, $CDCl_3$): 7.68-7.80 (m, 5H, Ar*H*), 2.50-2.73 (m, 4H), 2.33-2.40 (m, 2H), 2.07-2.19 (m, 2H), 1.67-1.80 (m, 4H), 1.30-1.44 (m, 4H), 0.85 (t, *J* = 7.1 Hz, CH_3 3H).^{13}C NMR (100 MHz, $CDCl_3$): 134.81 (d, J = 2.9 Hz), 131.44 (d, J = 8.9 Hz), 130.66 (d, J = 12.0 Hz) 119.93 (q, *J* = 321.8 Hz), 116.70 (d, J = 80.0 Hz), 24.55 (d, *J* = 6.7 Hz), 23.40 (d, *J* = 516.1 Hz), 23.03 (d, *J* = 4.7 Hz), 22.68 (d, *J* = 48.1 Hz), 20.90 (d, *J* = 6.5 Hz), 17.37 (d, *J* = 48.3 Hz), 13.15. ^{31}P (161 MHz, $CDCl_3$): 20.54. Calculated for $C_{17}H_{24}NO_4S_2PF_6$: C, 39.61%; H, 4.69%; N, 2.72%. Found: C, 39.96%; H, 5.01%; N, 3.03%.

Physical Measurements - Water contents of the ionic liquid samples were determined using a Mettler Toledo DL39 coulometric Karl Fischer titrator connected to an analytical balance. The water contents of the samples used for the property measurements ranged from 76–155 ppm.

Viscosities were measured with a Cambridge Applied Systems ViscoLab 4100 electromagnetic reciprocating piston viscometer that was temperature regulated by a Lauda RM-6 recirculating water bath. Viscosities for each of the ILs were recorded at intervals between 9 °C and 90 °C in ascending and descending order within their liquid ranges.

Variable temperature ionic conductivities were determined by AC impedance spectroscopy over the frequency range of 1–106 Hz, using a Solartron SI 1260 Impedance Phase Gain Analyzer. Equilibration times of 25–30 minutes between temperature change. 1 M KCl was used as the reference. Ionic conductivity values given are the average of two measurements. The values have uncertainties of 7%.

Thermal profiles: A TA Instruments Q100 Differential Scanning Calorimeter (DSC) was used to determine melting points and glass transition onset temperatures (T_g) and a TA Instruments Q500 Thermogravimetric Analyzer (TGA) was used for the determination of thermal decomposition profiles under nitrogen atmosphere. Scan rates were 5 °C/min for DSC and 10 °C/min for TGA. The data were analyzed using TA Universal Analysis 2000 software.

Self-diffusion coefficients: ^{1}H and ^{19}F NMR spectra, spin–lattice relaxation (T_1) times, and self-diffusion coefficients (D) were determined at the ^{1}H Larmor frequency of 300 MHz on a Varian Direct Digital Drive spectrometer with a 7.1

Tesla superconducting magnet. NMR spectra were obtained from the Fourier Transform of single π/2 pulses. *D* values were obtained by the Pulsed Gradient Spin Echo (PGSE) technique [17], and gradient values g ranged from 0.5–210 T/m. Spin–lattice relaxation times (T_1) were determined by the Inversion Recovery (π-τ-π/2-acquire) pulse sequence. All measurements were done on a 5 mm Doty dual frequency diffusion probe. Materials investigated were butylmethylphospholanium NTf_2, butylmethylphosphinanium NTf_2, and butylphenylphosphinanium NTf_2. Samples were packed under argon into 5 mm NMR sample tubes. Measurements were done at selected temperatures between 20–100 °C with 15–30 min equilibration times. The *D* values have uncertainties of 5%.

References

1. Fox, R. B. *J. Am. Chem. Soc.* **1950,** *72,* 4147–4149. doi:10.1021/ja01165a085
